# Correction to “FABP4 Mediates Inflammation to Regulate Endometrial Epithelial Cell Function During Early Gestation in Sheep”

**DOI:** 10.1002/vms3.70414

**Published:** 2025-05-30

Chen, Y., C. Liu, Z. Liu, et al. 2025. “FABP4 Mediates Inflammation to Regulate Endometrial Epithelial Cell Function During Early Gestation in Sheep.” *Veterinary Medicine and Science* 11, no. 2: e70193. https://doi.org/10.1002/vms3.70193.

In the correspondence section, Guangdong Hu was inadvertently removed and should have been included as one of the corresponding authors.

The correct correspondence should read: Xiayu Peng (pengxiayu@163.com), Jing Wang (wjtry100@163.com), and Guangdong Hu (guangdonghu@shzu.edu.cn).

We apologize for this error.

